# Synthesis and Determination of Physicochemical Properties of New 3-(4-Arylpiperazin-1-yl)-2-hydroxypropyl 4-Alkoxyethoxybenzoates

**DOI:** 10.3390/molecules21121682

**Published:** 2016-12-07

**Authors:** Pavlina Marvanova, Tereza Padrtova, Klara Odehnalova, Ondrej Hosik, Michal Oravec, Petr Mokry

**Affiliations:** 1 Department of Chemical Drugs, Faculty of Pharmacy, University of Veterinary and Pharmaceutical Sciences, Palackeho 1, 61242 Brno, Czech Republic; padrtova.tereza@seznam.cz (T.P.); odehnalovak@vfu.cz (K.O.); f12058@vfu.cz (O.H.); 2 Global Change Research Institute CAS, Belidla 986/4a, 60300 Brno, Czech Republic; oravec.m@czechglobe.cz

**Keywords:** arylcarbonyloxyaminopropanols, phenylpiperazines, p*K*_a_ determination, lipophilicity index, Lipinski rules, blood–brain barrier

## Abstract

Nine new dihydrochloride salts of 3-(4-arylpiperazin-1-yl)-2-hydroxypropyl 4-alkoxyethoxybenzoates were designed and synthesized. The physicochemical properties such as lipophilicity index (log *k_w_*) and dissociation constant (p*K*_a_) were experimentally determined and compared to the software calculated data. The lipophilicity index was determined by means of reversed-phase high performance liquid chromatography (RP-HPLC). The p*K*_a_ values were determined by means of capillary zone electrophoresis. The “drug-likeness” properties according to the Lipinski Rule of Five and prediction of possible blood–brain barrier penetration were computed and discussed.

## 1. Introduction

Nowadays, many drugs used in clinical practice have acidic or basic functionalities in their structures, antagonists of β-adrenergic receptors (β-blockers) included. β-blockers, one of the most prescribed and used cardiovascular drugs for more than 50 years, are still being improved with additional features such as vasodilatory and antioxidant activity. The dual α_1_- and β-blockers (e.g., carvedilol, labetalol, vanylidilol) showed hemodynamic benefits, including reduction of peripheral vascular resistance, improvement of cardiac output and left ventricular function, which is why the vasodilatory activity is an enhancement to the antihypertensive effect of β-blockade and offsets its drawback [1,2,3]. The pharmacokinetic effect and toxicity of the newly prepared compound can be predicted also from the determination of their physicochemical properties [4,5].

The physicochemical parameters such as lipophilicity (log *P*), distribution coefficient (log *D*) and dissociation constant (p*K*_a_) of an active pharmaceutical ingredient belong to crucial properties used to estimate the absorption, distribution, metabolism, and excretion (ADME) of a compound in biological systems as well as quantitative structure-activity and structure property relationship (QSAR/QSPR) profiling [6,7,8]. There are a number of commercially available programs that can predict these values, but the results of prediction can considerably differ depending on the algorithm used [9]. Therefore, predicted values should be replaced by experimental data in early stage of drug development. Among others, the reversed-phase high performance liquid chromatography (RP-HPLC) method is a successfully used tool for lipophilicity assessment [10,11,12]. One of the most predictive methods for lipophilicity determination is realized by monitoring the logarithmic retention factor (log *k_i_*) of the analyte with changing ratio of organic mobile phase fraction. Isocratic retention factors are extrapolated to 100% water to obtain log $k_{w}^{app}$ values at a given pH. The dissociation constant p*K*_a_ can be commonly determined by means of potentiometric titration, UV-VIS spectroscopy, capillary electrophoresis (CE), RP-HPLC or NMR spectroscopy [13,14]. The determination by means of CE proved to be a suitable technique [15,16,17], where the p*K*_a_ values are determined from monitoring dependence of effective mobility of the analyte on pH of the background electrolyte (BGE) using non-linear regression analysis.

This contribution is focused on the synthesis and experimental determination of the physicochemical properties of nine new 3-(4-arylpiperazin-1-yl)-2-hydroxypropyl 4-alkoxyethoxybenzoates. The benzene ring (a part of 4-hydroxybenzoic acid) in an arylcarbonyloxyaminopropanol fragment is substituted in the C4 position with a propoxyethoxy, isopropoxyethoxy or allyloxyethoxy chain. The basic part of the molecule is formed with phenylpiperazine fragment that is either unsubstituted or substituted with a methoxy moiety in the C2’ or C4’ position. All the compounds were prepared as dihydrochloride salts to increase their solubility in water. The newly synthesized agents are expected to have cardiovascular activity due to the presence of particular structures of pharmacophores (arylcarbonyloxyaminopropanol moiety responsible for β-adrenolytic activity [18], phenylpiperazine fragment providing α_1_-adrenolytic activity [19]), nevertheless, the biological activities of the discussed compounds have not been determined so far.

## 2. Results and Discussion

### 2.1. Chemistry

**Scheme 1:**
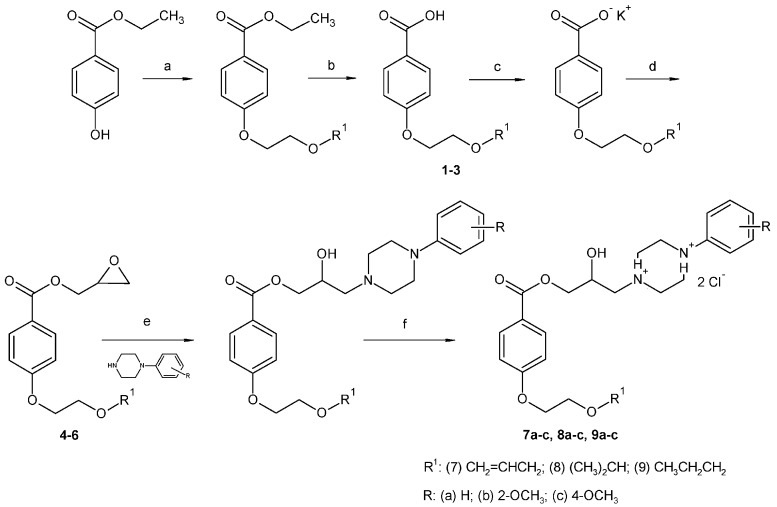
Synthesis of discussed compounds **1**–**9a**–**c**. *Reagents and conditions*: (**a**) **T1a**–**c**, K_2_CO_3_, acetone, reflux; (**b**) NaOH, HCl, reflux; (**c**) KOH, MeOH, propan-2-ol (IPA); (**d**) **T2**; dimethylformamide (DMF), reflux; (**e**) arylpiperazine, IPA, reflux; (**f**) HCl, Et_2_O.

**Scheme 2:**
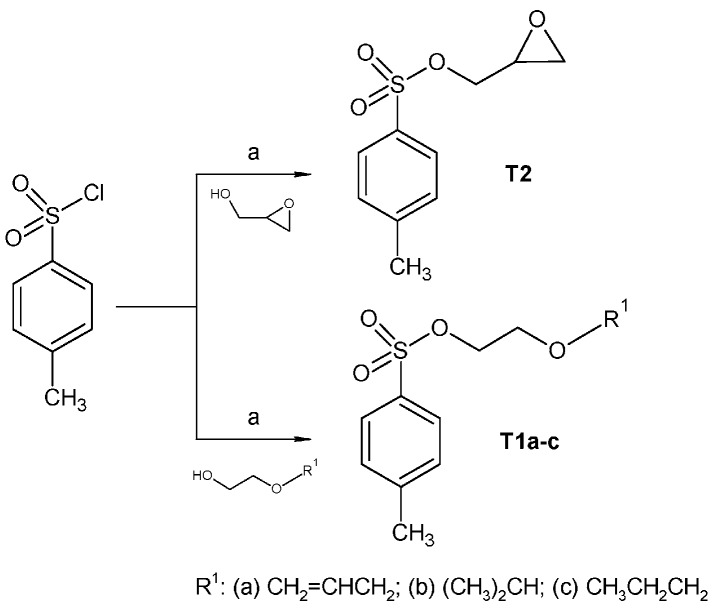
Synthesis of intermediates **T1a**–**c** and **T2**. *Reagents and conditions*: (**a**) TEA, DCM, 0 °C.

The final products were prepared via multi-step synthesis as described in Scheme 1. Tosylate intermediates **T1a**–**c** and **T2** were prepared according to the published procedure (Scheme 2), and the spectroscopic and physical data were in agreement with those reported in the literature [20,21,22,23]. The reaction temperature (0 °C) and time (two to three hours) was crucial to obtain pure products at good yields. Intermediates **1**–**3** were already described in the literature but were prepared by a different synthetic method, and their spectroscopic data were not published yet. Intermediates **4**–**6** were prepared from ethyl 4-hydroxybenzoate via reaction with the appropriate tosylate intermediates **T1a**–**c**. The reaction was carried out in acetone with potassium carbonate at 65 °C for 12 h and then stirred for at least 12 h at ambient temperature, changing the solvent (acetonitrile, dimethylformamide) did not improve the purity nor the yield of the desired products. The hydrolysis was then performed by using a 2 M solution of sodium hydroxide and the desired acids **4**–**6** were obtained by displacing with HCl, extracting to chloroform and evaporating. The final products, hydrochloride salts **7a**–**c**, **8a**–**c**, **9a**–**c**, were prepared according to the already published synthesis [24]. Briefly, the appropriate acids **1–3** were transformed into the potassium salt using potassium hydroxide in a mixture of methanol and propan-2-ol (ratio 1:3). Compounds **4**–**6**, (oxiran-2-yl)methyl 4-(2-alkoxyethoxy)benzoates were obtained by reaction of intermediate **T2** and potassium 4-(2-alkoxyethoxy)benzoate in dimethylformamide for 7 h at 70 °C. The oxirane ring was then opened by appropriate phenylpiperazine, the reaction was carried out in propan-2-ol for one hour at 80 °C and for 72 h at ambient temperature. The obtained bases were finally converted to their dihydrochloride salts by extend of hydrochloric acid to enhance the solubility of the compounds in water. The protonation of the phenylpiperazine nitrogens was not experimentally determined for the discussed final compounds but the hypothesis is based on the measurement carried out for similar structures [24].

### 2.2. Physicochemical Properties

The dissociation constants (p*K*_a_) of studied compounds were determined by means of CE. The measured values and values predicted by chemical software (ACD Percepta ver. 2012, Advanced Chemistry Development, Toronto, ON, Canada; MarvinSketch 6.0.6., ChemAxon Kft., Budapest, Hungary) are shown in Table 1. The correlation coefficients of the experimentally determined values were greater than 0.999 and standard deviation equal or less than 0.02 units (*n* = 4). The experimental values are in the range 6.35–6.92 and the highest values were recorded for the 1-(2-methoxyphenylpiperazine) derivatives **7b**, **8b**, **9b**. The clinically used β-blockers are usually stronger bases [14], the values of studied compounds are lower due to the presence of piperazine moiety instead of secondary amine group.

The lipophilicity indexes log $k_{w}^{7.4}$ were measured by means of RP-HPLC method. The log *k_w_* values were then calculated according to Equation (2) given in Section 3.3. The correlation coefficients were greater than 0.999 with covariance equal or less than 0.03 units (*n* = 2). All the measured, calculated and predicted values are shown in Table 2.

The lipophilicity decreases in order from non-substituted through *ortho*- to *para*-substituted derivatives, and also in order from propoxy- through isopropoxy- to allyloxy-derivatives.

Moreover, other parameters, such as number of hydrogen bond donors (nOHNH), number of hydrogen bond acceptors (nON), number of rotatable bonds (nRTB) and a polar surface area (PSA) known as Lipinski criteria as well as blood–brain barrier (BBB) penetration (log BB), were estimated and evaluated. All these parameters influence the toxicity and ADME of a drug in human body. There is a great emphasis on the prediction of drug penetration through BBB in order to avoid complications in the later phases of drug development due to undesirable neurotoxicity as a side effect [25]. The penetration through the BBB has been negatively related to molecular properties such as high drug lipophilicity, molecular weight, and the tendency to form hydrogen bonds quantified as PSA [26,27,28]. As shown in Table 3, the discussed compounds do fulfill the Lipinski rules, except the number of rotatable bonds, but the already clinically-used landiolol shows similarities. A large number of rotatable bonds has been associated with poor oral bioavailability, particularly when associated with a high polar surface area [29]. For prediction of the ability of our compounds to cross the BBB by passive transport, we chose the model proposed by Vilar et al. [27]. Based on previously described facts that compounds with log BB > 0.3 pass the BBB readily and compounds with log BB < −1 are poorly distributed in the brain [30,31], Vilar et al. generated two distinct classification models for both threshold values. These models were generated using in vivo log BB values of 307 compounds and validated on 1457 molecules using their in silico physicochemical descriptors with the percentage of good classified compounds around 80% and can be used also for prediction of log BB values of novel compounds. According to the results calculated by model 1 (results shown in Table 3) none of our compounds exceeded the threshold value of 0.3, suggesting that these compounds could not cross the BBB readily. However the values calculated according to the model 2, which suggests poor penetration through the BBB, are greater than the threshold value (log BB > −1). Therefore, studied drugs belong to the category of compounds with log BB values comprised between 0.3 and −1, which still have access to the central nervous system (CNS); i.e., the studied compounds can possess low brain permeability. These results should be taken into account during the further stages of drug development.

## 3. Experimental Section

### 3.1. General Information

All reagents were purchased from Sigma-Aldrich (St. Louis, MO, USA) or Acros Organics (Geel, Belgium) in sufficient purity. The solvents were purchased from Lach-Ner (Neratovice, Czech Republic) and were dried or freshly distilled if necessary. Thin layer chromatography (TLC) Kieselgel 60 F_254_ plates (Merck, Darmstadt, Germany) visualized by UV irradiation (254 nm) were used to monitor the reactions and the purity of the prepared substances; the reversed-phase TLC RP-18 F_254_ plates (Merck) were used for the final compounds. The melting points were determined on a Kofler hot-plate apparatus HMK (Franz Kustner Nacht KG, Dresden, Germany) and are uncorrected. The purity of final compounds was analysed by a Dionex Ultimate 3000 (Thermo Fisher Scientific, Waltham, MA, USA) HPLC system controlled through the Chromeleon^®^ Chromatography Data System (version 7.2, Thermo Fisher Scientific). The separation was performed on ZORBAX Extend-C_18_ (3.5 µm, 3 × 150 mm) column (Agilent Technologies, Waldbronn, Germany). Mobile phase consisted of 0.02 M 3-morpholinopropanesulphonate buffer with addition of 0.15% of *n*-decylamine (pH = 7.4; constituent A) and methanol to which 0.25% of *n*-octanol was added (constituent B). The total flow rate was 0.4 mL/min, the injection volume was 1 μL, and the column temperature was maintained at 25 °C. The detection wavelength of 254 nm was chosen. The purity of individual compounds was calculated as the average of relative peak areas in the chromatograms of the sample solution under isocratic conditions in the range 25:75–50:50 (A:B; *v/v*). The IR spectra were recorded on a Nicolet Impact 410 FT-IR spectrometer (Thermo Fisher Scientific) using Attenuated Total Reflection (ATR, ZnSe) instrumentation. The spectra were obtained by accumulation of 32 scans in the region 4000–600 cm^−^^1^. The instrument was controlled by Omnic v. 8.3 software (Thermo Fisher Scientific). The following abbreviations are used for the characteristic infrared bands: ν(O–H), alcohol O–H stretching vibration band; ν(C–H), C–H stretching vibration bands; ν(NH^+^), hydrochloride salt of piperazine N-H stretching vibration band; ν(C=O), ester C=O stretching vibration band; ν(arom. C=C), aromatic ring C=C stretching vibration band; ν(C–N), piperazine C-N stretching vibration band; ν(C–O–C), ether C–O stretching vibration band. The 1D (^1^H, ^13^C{^1^H}, ^13^C-APT) and 2D (^1^H-^1^H COSY, ^1^H-^13^C HMQC and ^1^H-^13^C HMBC) NMR experiments for characterization of the final compounds were performed using a JNM-ECZ4OOR FT-NMR spectrometer 9.39 T (399.78 MHz for ^1^H and 100.53 MHz for ^13^C nucleus; JEOL RESONANCE Inc., Tokyo, Japan) equipped with a 5 mm High Sensitivity Pulse Field Gradient Autotune™ probe. Chemical shifts are reported in ppm, referenced to the chemical shifts of residual solvent resonance (DMSO, 2.5 ppm for ^1^H and 39.5 ppm for ^13^C). The coupling constants (*J*) are reported in Hz. The NMR spectra of intermediates were measured in DMSO-*d*_6_ on a Gemini-2000 FT-NMR spectrometer (200 MHz for ^1^H and 50 MHz for ^13^C, Varian Comp., Palo Alto, CA, USA). The following abbreviations are used to describe the NMR signal multiplicities: s, singlet; d, doublet; t, triplet; q, quartet; dd, doublet of doublet; dt, doublet of triplet; dq, doublet of quartet; ddt, doublet of doublet of triplet; m, multiplet.

High-resolution mass spectra of the final compounds were measured using a high-performance liquid chromatograph Dionex UltiMate^®^ 3000 (Thermo Fisher Scientific, West Palm Beach, FL, USA) coupled with a LTQ Orbitrap XL™ Hybrid Ion Trap-Orbitrap Fourier Transform Mass Spectrometer (Thermo Scientific) with injection into HESI II in the negative mode.

### 3.2. Synthesis

#### 3.2.1. Synthesis of Tosylate Intermediates

*p*-Toluensulfonyl chloride (2.6 g, 0.014 mol) was slowly added to the mixture of appropriate 2-alkoxyethanol respectively (oxiran-2-yl)methanol (0.014 mol) and triethylamine (1.84 g, 0.018 mol) in dichlormethane (20 mL) cooled at 0 °C. The mixture was then stirred for 1 h at ambient temperature. The precipitate was filtered and the organic phase was washed with 2 M HCl, saturated solution of Na_2_CO_3_ and water, dried over MgSO_4_, and then the solvent was evaporated. Product **T2** was recrystallized from petroleum ether/diethyl ether in ratio 5:1.

*Allyloxyethyl 4-methylbenzensulfonate* (**T1a**). Yield: 97%; *R*_f_: 0.74 (ethyl acetate/petroleum ether 1:1); ^1^H-NMR (200 MHz, DMSO-*d*_6_) δ (ppm): 7.79 (d, *J* = 8.5 Hz, 2H, Ar-H^2,6^), 7.48 (d, *J* = 8.5 Hz, 2H, Ar-H^3,5^), 5.79 (ddt, *J* = 17.2, 10.4, 5.4 Hz, 1H, -CH=), 5.27–5.07 (m, 2H, =CH_2_), 4.15–4.11 (m, 2H, TsOCH_2_), 3.87 (dt, *J* = 5.3, 1.4 Hz, 2H, -CH_2_CH=), 3.56–3.52 (m, 2H, CH_2_CH_2_O), 2.42 (s, 3H, ArCH_3_); ^13^C-NMR (50 MHz, DMSO-*d*_6_) δ (ppm): 144.77, 134.62, 132.40, 130.00, 127.47, 116.47, 70.78, 69.84, 66.93, 20.98. (CAS Registry Number 50563-72-9).

*Isopropoxyethyl 4-methylbenzensulfonate* (**T1b**). Yield: 85%; *R*_f_: 0.70 (ethyl acetate/petroleum ether 1:1); ^1^H-NMR (200 MHz, DMSO-*d*_6_) δ (ppm): 7.78 (d, *J* = 8.5 Hz, 2H, Ar-H^2,6^), 7.48 (d, *J* = 8.5, 2H, Ar-H^3,5^), 4.10–4.06 (m, 2H, TsOCH_2_), 3.54–3.41 (m, 3H, CH_2_CH_2_O + -CH(CH_3_)_2_), 2.42 (s, 3H, ArCH_3_), 1.00 (d, *J* = 6.0, 6H, -CH(C**H_3_**)**_2_**); ^13^C-NMR (50 MHz, DMSO-*d*_6_) δ (ppm): 144.82, 132.47, 130.06, 127.57, 70.91, 70.44, 64.85, 21.76, 21.05. (CAS Registry Number 51218-98-5).

*Propoxyethyl 4-methylbenzensulfonate* (**T1c**). Yield: 86%; *R*_f_: 0.72 (ethyl acetate/petroleum ether 1:1); ^1^H-NMR (200 MHz, DMSO-*d*_6_) δ (ppm): 7.78 (d, *J* = 8.5 Hz, 2H, Ar-H^2,6^), 7.48 (d, *J* = 8.5, 2H, Ar-H^3,5^), 4.13–4.09 (m, 2H, TsOCH_2_), 3.53–3.49 (m, 2H, CH_2_CH_2_O), 3.23 (t, *J* = 6.4 Hz, 2H, -CH_2_CH_2_CH_3_), 2.42 (s, 3H, ArCH_3_), 1.50–1.32 (m, 2H, -CH_2_CH_2_CH_3_), 0.77 (t, *J* = 7.7 Hz, 3H, -CH_3_); ^13^C-NMR (50 MHz, DMSO-*d*_6_) δ (ppm): 144.85, 132.43, 130.08, 127.57, 71.71, 70.00, 67.39, 22.22, 21.05, 10.32. (CAS Registry Number 52497-47-9).

*(Oxiran-2-yl)methyl 4-methylbenzensulfonate* (**T2**). Yield: 97%; *R*_f_: 0.77 (ethyl acetate/petroleum ether 1:1); m.p.: 35–37 °C; ^1^H-NMR (200 MHz, DMSO-*d*_6_) δ (ppm): 7.80 (d, *J* = 8.4 Hz, 2H, Ar-H^2,6^), 7.49 (d, *J* = 8.4 Hz, 2H, Ar-H^3,5^), 4.41 (dd, *J* = 11.5, 2.4 Hz, 1H, TsOCH_2_), 3.81 (dd, *J* = 11.5, 7.1 Hz, 1H, TsOCH_2_), 3.22–3.14 (m, 1H, CH-oxiran), 2.79–2.74 (m, 1H, CH_2_-oxiran), 2.60 (dd, *J* = 5.0, 2.6 Hz, 1H, CH_2_-oxiran), 2.42 (s, 3H, ArCH_3_); ^13^C-NMR (50 MHz, DMSO-*d*_6_) δ (ppm): 144.99, 132.20, 130.10, 127.51, 71.58, 48.49, 43.63, 20.98. (CAS Registry Number 118712-54-2).

#### 3.2.2. Synthesis of 4-Alkoxyethoxybenzoic Acid

A mixture of ethyl-4-hydroxybenzoate (3.32 g, 0.02 mol), tosylate intermediate **T1a**–**c** (0.02 mol) and potassium carbonate (0.06 mol) in acetone (25 mL) was heated at 70 °C for 8 h and then stirred for 12 h at room temperature. The reaction was monitored by means of TLC. The precipitation was then filtered and acetone was evaporated. The residue was dissolved in ethyl acetate and washed with 2 M NaOH and water, dried over MgSO_4_, and then the solvent was evaporated. The crude product heated for 2 h with 5 M NaOH (50 mL) at 100 °C. The reaction mixture was washed with chloroform and neutralized with concentrated HCl. The resulting white precipitate was collected by filtration.

*4-(2-Allyloxy)ethoxybenzoic acid* (**3a**). Yield: 69%; *R*_f_: 0.67 (ethyl acetate); m.p.: 120–123 °C; ^1^H-NMR (200 MHz, DMSO-*d*_6_) δ (ppm): 12.60 (bs, 1H, COOH), 7.88 (d, *J* = 9.0 Hz, 2H, Ar-H^2,6^), 7.02 (d, *J* = 9.0 Hz, 2H, Ar-H^3,5^), 5.89 (ddt, *J* = 17.3, 10.5, 5.3 Hz, 1H, -CH=), 5.26 (dq, *J* = 17.3, 1.8 Hz, 1H, =CH_2_), 5.18–5.11 (m, 1H, =CH_2_), 4.20–4.16 (m, 2H, ArOCH_2_), 4.01 (dt, *J* = 5.3, 1.6 Hz, 2H, -CH_2_CH=), 3.75–3.71 (m, 2H, CH_2_O_allyl_); ^13^C-NMR (50 MHz, DMSO-*d*_6_) δ (ppm): 166.94, 162.05, 135.03, 131.30, 123.03, 116.52, 114.25, 71.10, 67.91, 67.37. (CAS Registry Number 153881-37-9).

*4-(2-Isopropoxy)ethoxybenzoic acid* (**3b**). Yield: 65%; *R*_f_: 0.67 (ethyl acetate); m.p.: 128–130 °C; ^1^H-NMR (200 MHz, DMSO-*d*_6_) δ (ppm): 12.61 (bs, 1H, COOH), 7.87 (d, *J* = 9.0 Hz, 2H, Ar-H^2,6^), 7.02 (d, *J* = 9.0 Hz, 2H, Ar-H^3,5^), 4.15–4.10 (m, 2H, ArOCH_2_), 3.72–3.56 (m, 3H, -CH_2_OCH(CH_3_)_2_), 1.10 (d, *J* = 6.0 Hz, 6H, -CH(CH_3_)_2_); ^13^C-NMR (50 MHz, DMSO-*d*_6_) δ (ppm): 166.96, 162.13, 131.30, 122.96, 114.26, 71.07, 67.79, 65.74, 21.99. (CAS Registry Number 766552-53-8).

*4-(2-Propoxy)ethoxybenzoic acid* (**3c**). Yield: 69%; *R*_f_: 0.70 (ethyl acetate); m.p.: 109–111 °C (lit. 114–115 °C); ^1^H-NMR (200 MHz, DMSO-*d*_6_) δ (ppm): 12.63 (bs, 1H, COOH), 7.85 (d, *J* = 9.0 Hz, 2H, Ar-H^2,6^), 7.02 (d, *J* = 9.0 Hz, 2H, Ar-H^3,5^), 4.18–4.13 (m, 2H, ArOCH_2_), 3.72–3.68 (m, 2H, -CH_2_OPr), 3.40 (t, *J* = 6.6 Hz, 2H, -CH_2_CH_2_CH_3_), 1.60–1.43 (m, 2H, -CH_2_CH_2_CH_3_), 0.85 (t, *J* = 7.5 Hz, 3H, -CH_2_CH_2_CH_3_); ^13^C-NMR (50 MHz, DMSO-*d*_6_) δ (ppm): 166.96, 162.10, 131.30, 122.99, 114.25, 72.01, 68.35, 67.44, 22.38, 10.44. (CAS Registry Number: 67132-01-8).

#### 3.2.3. Synthesis of Oxirane-Intermediates

A mixture of 4-alkoxyethoxybenzoic acid **3a–c** (0.028 mol) in methanol (75 mL) and KOH (2.2 g, 0.042 mol) in propan-2-ol (50 mL) was stirred for 1 h at room temperature, and after that, propan-2-ol (175 mL) was added for the final ratio methanol/propan-2-ol 1:3. The resulting white precipitate was collected by filtration and dried under low pressure. A mixture of appropriate potassium salt (5 g, 0.023 mol) with (oxiran-2-yl)methyl 4-methylbenzensulfonate **T2** (3.5 g, 0.015 mol) in DMF (50 mL) was heated for 7 h at 70 °C. The reaction was monitored by means of TLC. The solvent was evaporated, and the residue was dissolved in ethyl acetate and washed with water; the organic layer was dried over MgSO_4_, and ethyl acetate was evaporated.

*(Oxiran-2-yl)methyl 4-allyloxyethoxybenzoate* (**4**). Yield: 88%; *R*_f_: 0.79 (ethyl acetate/petroleum ether 1:1); ^1^H-NMR (200 MHz, DMSO-*d*_6_) δ (ppm): 7.93 (d, *J* = 8.9 Hz, 2H, Ar-H^2,6^), 7.07 (d, *J* = 8.9 Hz, 2H, Ar-H^3,5^), 5.90 (ddt, *J* = 17.3, 10.5, 5.3 Hz, 1H, -CH=), 5.27 (dq, *J* = 17.4, 1.8 Hz, 1H, =CH_2_), 5.15 (dq, *J* = 10.5, 1.5, 1H, =CH_2_), 4.60 (dd, *J* = 12.4, 2.7 Hz, 1H, COOCH_2_), 4.21–4.19 (m, 2H, ArOCH_2_), 4.08–4.01 (m, 3H, COOCH_2_ + -CH_2_CH=), 3.75–3.73 (m, 2H, CH_2_O_allyl_), 3.34–3.30 (m, 1H, CH-oxirane), 2.84–2.82 (m, 1H, CH_2_-oxirane), 2.73–2.71 (m, 1H, CH_2_-oxirane); ^13^C-NMR (50 MHz, DMSO-*d*_6_) δ (ppm): 165.07, 162.54, 135.02, 131.34, 121.51, 116.51, 114.52, 71.09, 67.87, 67.46, 65.05, 49.07, 43.83.

*(Oxiran-2-yl)methyl 4-isopropoxyethoxybenzoate* (**5**). Yield: 84%; *R*_f_: 0.78 (ethyl acetate/petroleum ether 1:1); ^1^H-NMR (200 MHz, DMSO-*d*_6_) δ (ppm): 7.92 (d, *J* = 8.7 Hz, 2H, Ar-H^2,6^), 7.06 (d, *J* = 8.7 Hz, 2H, Ar-H^3,5^), 4.60 (dd, *J* = 12.4, 2.5 Hz, 1H, COOCH_2_), 4.16–4.14 (m, 2H, ArOCH_2_-), 4.05 (dd, *J* = 12.4, 6.4 Hz, 1H, COOCH_2_), 3.72–3.69 (m, 2H, -CH_2_OiPr), 3.67–3.58 (m, 1H, -CH(CH_3_)_2_), 3.34–3.30 (m, 1H, CH-oxirane), 2.84–2.82 (m, 1H, CH_2_-oxirane), 2.73–2.71 (m, 1H, CH_2_-oxirane), 1.10 (d, *J* = 6.0 Hz, 6H, -CH(CH_3_)_2_); ^13^C-NMR (50 MHz, DMSO-*d*_6_) δ (ppm): 165.10, 162.62, 131.35, 121.46, 114.55, 71.09, 67.88, 65.71, 65.08, 49.09, 43.84, 21.98.

*(Oxiran-2-yl)methyl 4-propoxyethoxybenzoate* (**6**). Yield: 87%; *R*_f_: 0.69 (ethyl acetate/petroleum ether 1:1); ^1^H-NMR (200 MHz, DMSO-*d*_6_) δ (ppm): 7.92 (d, *J* = 8.7 Hz, 2H, Ar-H^2,6^), 7.07 (d, *J* = 8.7 Hz, 2H, Ar-H^3,5^), 4.60 (dd, *J* = 12.4, 2.7 Hz, 1H, COOCH_2_), 4.19–4.16 (m, 2H, ArOCH_2_), 4.05 (dd, *J* = 12.4, 6.4 Hz, 1H, COOCH_2_), 3.72–3.70 (m, 2H, -CH_2_OPr), 3.40 (t, *J* = 6.6 Hz, 2H, -CH_2_CH_2_CH_3_), 3.34–3.30 (m, 1H, CH-oxirane), 2.84–2.82 (m, 1H, CH_2_-oxirane), 2.73–2.71 (m, 1H, CH_2_-oxirane), 1.56–1.47 (m, 2H, -CH_2_CH_2_CH_3_), 0.85 (t, *J* = 7.3 Hz, 3H, -CH_2_CH_2_CH_3_); ^13^C-NMR (50 MHz, DMSO-*d*_6_) δ (ppm): 165.10, 162.60, 131.35, 121.49, 114.53, 72.03, 68.33, 67.54, 65.08, 49.09, 43.84, 22.38, 10.44.

#### 3.2.4. Synthesis of Final Compounds

Intermediate **4**–**6** (0.004 mol) was added to the solution of the corresponding arylpiperazine (0.004 mol) in propan-2-ol (15 mL). The reaction mixture was heated at 80 °C for 1 h and stirred for 72 h at room temperature. After that, the reaction mixture was cooled at least for 48 h at −18 °C. Precipitating crude base was filtered and dissolved in diethyl ether or chloroform and transformed to their dihydrochloride salt **7a**–**c**, **8a**–**c**, **9a**–**c** by addition of an excess of saturated ethereal hydrogen chloride. The precipitate was filtered and recrystallized from propan-2-ol, if necessary.

*1-{3-[(4-Allyloxyethoxybenzoyl)oxy]-2-hydroxypropyl}-4-phenylpiperazinediium dichloride* (**7a**). Yield: 23%; *R*_f_: 0.87 (acetone/toluene 3:1); *R*_f_ reverse-phase (rev.): 0.51 (0.1 M HCl/acetone 3:2); m.p.: 185–187 °C; HPLC purity (pur.) 91.67% (254 nm); IR (ATR, cm^−1^): ν(O–H) = 3289, ν(C–H) = 2930, ν(NH^+^) = 2537, ν(C=O) = 1697, ν(arom. C=C) = 1603, ν(C–N) = 1248, ν(C–O–C) = 1175; ^1^H-NMR (399.78 MHz, DMSO-*d*_6_) δ (ppm): 10.75 (bs, 2H, NH^+^_pip_), 8.00 (d, *J* = 8.7 Hz, 2H, H_ArCOO_), 7.28–7.24 (m, 2H, H_Ar-pip_), 7.07 (d, *J* = 8.7 Hz, 2H, H_ArO_), 7.01–6.97 (m, 2H, H_Ar-pip_), 6.88–6.84 (m, 1H, H_Ar-pip_), 5.90 (ddt, *J* = 17.4, 10.5, 5.4 Hz, 1H, -CH=), 5.27 (dq, *J* = 17.4, 1.8 Hz, 1H, =CH_2_), 5.16 (dq, *J* = 10.5, 1.8 Hz, 1H, =CH_2_), 4.52–4.45 (m, 1H, CH_OH_), 4.23–4.20 (m, 4H, COOCH_2_ + ArOCH_2_), 4.02 (dt, *J* = 5.4, 1.4 Hz, 2H, -CH_2_CH=), 3.84–3.61 (m, 6H, H_pip_ + CH_2_O_allyl_), 3.44–3.15 (m, 6H, H_pip_ + CH_2_N_pip_); ^13^C-NMR (100.53 MHz, DMSO-*d*_6_) δ (ppm): 165.21, 162.57, 149.60, 135.06, 131.62, 129.14, 121.67, 119.93, 116.62, 115.89, 114.43, 71.13, 67.92, 67.50, 66.18, 63.33, 58.13, 52.13, 50.75, 45.25, 45.14; HR-MS: C_25_H_33_N_2_O_5_ [M − H]^−^ calculated 441.2384 *m*/*z*, found 441.2383 *m*/*z*.

*1-{3-[(4-Allyloxyethoxybenzoyl)oxy]-2-hydroxypropyl}-4-(2-methoxyphenyl)piperazinediium dichloride* (**7b**). Yield: 39%; *R*_f_: 0.83 (acetone/toluene 3:1); *R*_f_ (rev.): 0.57 (0.1 M HCl/acetone 3:2); m.p.: 178–180 °C; HPLC pur. 89.14% (254 nm); IR (ATR, cm^−1^): ν(O–H) = 3318, ν(C–H) = 2967, ν(NH^+^) = 2351, ν(C=O) = 1711, ν(arom. C=C) = 1603, ν(C–N) = 1252, ν(C–O–C) = 1167; ^1^H-NMR (399.78 MHz, DMSO-*d*_6_) δ (ppm): 10.86 (bs, 2H, NH^+^_pip_), 8.00 (d, *J* = 8.7 Hz, 2H, H_ArCOO_), 7.07 (d, *J* = 8.7 Hz, 2H, H_ArO_), 7.04–7.02 (m, 1H, H_Ar-pip_), 7.00–6.97 (m, 2H, H_Ar-pip_), 6.93–6.89 (m, 1H, H_Ar-pip_), 5.89 (ddt, *J* = 17.4, 10.5, 5.4 Hz, 1H, -CH=), 5.27 (dq, *J* = 17.4, 1.8 Hz, 1H, =CH_2_), 5.15 (dq, *J* = 10.5, 1.8 Hz, 1H, =CH_2_), 4.52–4.45 (m, 1H, CH_OH_), 4.22–4.19 (m, 4H, COOCH_2_ + ArOCH_2_), 4.01 (dt, *J* = 5.4, 1.4 Hz, 2H, -CH_2_CH=), 3.80 (s, 3H, OCH_3_), 3.77–3.60 (m, 6H, H_pip_ + CH_2_O_allyl_), 3.52–3.13 (m, 6H, H_pip_ + CH_2_N_pip_); ^13^C-NMR (100.53 MHz, DMSO-*d*_6_) δ (ppm): 165.22, 162.57, 151.85, 139.00, 135.08, 131.65, 123.79, 121.70, 120.88, 118.41, 116.62, 114.44, 112.03, 71.14, 67.93, 67.51, 66.18, 63.36, 58.35, 55.44, 52.50, 51.25, 46.90, 46.74; HR-MS: C_26_H_35_N_2_O_6_ [M − H]^−^ calculated 471.2489 *m*/*z*, found 471.2484 *m*/*z*.

*1-{3-[(4-Allyloxyethoxybenzoyl)oxy]-2-hydroxypropyl}-4-(4-methoxyphenyl)piperazinediium dichloride* (**7c**). Yield: 44%; *R*_f_: 0.82 (acetone/toluene 3:1); *R*_f_ (rev.): 0.58 (0.1 M HCl/acetone 3:2); m.p.: 170–173 °C; HPLC pur. 94.28 (254 nm); IR (ATR, cm^−1^): ν(O–H) = 3335, ν(C–H) = 2978, ν(NH^+^) = 2377, ν(C=O) = 1710, ν(arom. C=C) = 1604, ν(C–N) = 1250, ν(C–O–C) = 1166; ^1^H-NMR (399.78 MHz, DMSO-*d*_6_) δ (ppm): 10.96 (bs, 2H, NH^+^_pip_), 8.00 (d, *J* = 8.7 Hz, 2H, H_ArCOO_), 7.10 (d, *J* = 9.1 Hz, 2H, H_Ar-pip_), 7.07 (d, *J* = 8.7 Hz, 2H, H_ArO_), 6.91 (d, 2H, *J* = 9.1 Hz, H_Ar-pip_), 5.89 (ddt, *J* = 17.3, 10.5, 5.3 Hz, 1H, -CH=), 5.27 (dq, *J* = 17.3, 1.7 Hz, 1H, =CH_2_), 5.15 (dq, *J* = 10.5, 1.7 Hz, 1H, =CH_2_), 4.51–4.46 (m, 1H, CH_OH_), 4.23–4.19 (m, 4H, COOCH_2_ + ArOCH_2_), 4.01 (dt, *J* = 5.3, 1.7 Hz, 2H, -CH_2_CH=), 3.79–3.60 (m, 6H, H_pip_ + CH_2_O_allyl_), 3.71 (s, 3H, OCH_3_), 3.53–3.25 (m, 6H, H_pip_ + CH_2_N_pip_); ^13^C-NMR (100.53 MHz, DMSO-*d*_6_) δ (ppm): 165.23, 162.57, 154.69, 142.13, 135.08, 131.64, 121.69, 118.78, 116.62, 114.56, 114.44, 71.14, 67.93, 67.51, 66.20, 63.46, 58.04, 55.32, 51.82, 50.62, 47.26, 47.19; HR-MS: C_26_H_35_N_2_O_6_ [M − H]^−^ calculated 471.2489 *m*/*z*, found 471.2485 *m*/*z*.

*1-{2-Hydroxy-3-[(4-isopropoxyethoxybenzoyl)oxy]propyl}-4-phenylpiperazinediium dichloride* (**8a**). Yield: 22%; *R*_f_: 0.85 (acetone/toluene 3:1); *R*_f_ (rev.): 0.25 (0.1 M HCl/acetone 3:2); m.p.: 200–202 °C; HPLC pur. 95.12% (254 nm); IR (ATR, cm^−1^): ν(O–H) = 3287, ν(C–H) = 2972, ν(NH^+^) = 2559, ν(C=O) = 1699, ν(arom. C=C) = 1603, ν(C–N) = 1252, ν(C–O–C) = 1180; ^1^H-NMR (399.78 MHz, DMSO-*d*_6_) δ (ppm): 10.69 (bs, 2H, NH^+^_pip_), 7.99 (d, *J* = 8.7 Hz, 2H, H_ArCOO_), 7.28–7.24 (m, 2H, H_Ar-pip_), 7.06 (d, *J* = 8.7 Hz, 2H, H_ArO_), 7.02–7.00 (m, 2H, H_Ar-pip_), 6.88–6.84 (m, 1H, H_Ar-pip_), 4.51–4.44 (m, 1H, CH_OH_), 4.23–4.21 (m, 2H, COOCH_2_, 4.17–4.14 (m, 2H, ArOCH_2_), 3.84–3.57 (m, 7H, -CH_2_OCH(CH_3_)_2_ + H_pip_), 3.43–3.15 (m, 6H, -CH_2_N_pip_ + H_pip_), 1.10 (d, *J* = 5.9 Hz, 6H, -CH(CH_3_)_2_); ^13^C-NMR (100.53 MHz, DMSO-*d*_6_) δ (ppm): 165.23, 162.64, 149.52, 131.62, 129.16, 121.62, 120.03, 115.95, 114.46, 71.13, 67.93, 66.18, 65.76, 63.33, 58.11, 52.14, 50.71, 45.31, 45.19, 22.03; HR-MS: C_25_H_36_N_2_O_5_ [M − H]^−^ calculated 443.2540 *m*/*z*, found 443.2535 *m*/*z*.

*1-{2-Hydroxy-3-[(4-isopropoxyethoxybenzoyl)oxy]propyl}-4-(2-methoxyphenyl)piperazinediium dichloride* (**8b**). Yield: 25%; *R*_f_: 0.82 (acetone/toluene 3:1); *R*_f_ (rev.): 0.29 (0.1 M HCl/acetone 3:2); m.p.: 154–156 °C; HPLC pur. 98.87% (254 nm); IR (ATR, cm^−1^): ν(O–H) = 3318, ν(C–H) = 2969, ν(NH^+^) = 2560, ν(C=O) = 1701, ν(arom. C=C) = 1603, ν(C–N) = 1251, ν(C–O–C) = 1166; ^1^H-NMR (399.78 MHz, DMSO-*d*_6_) δ (ppm): 10.67 (bs, 2H, NH^+^_pip_), 7.99 (d, *J* = 8.7 Hz, 2H, H_ArCOO_), 7.07 (d, *J* = 8.7 Hz, 2H, H_ArO_), 7.03–6.89 (m, 4H, H_Ar-pip_), 4.49–4.44 (m, 1H, CH_OH_), 4.23–4.21 (m, 2H, COOCH_2_), 4.17–4.15 (m, 2H, ArOCH_2_), 3.80 (s, 3H, OCH_3_), 3.72–3.70 (m, 2H, -CH_2_O*i*Pr), 3.68–3.40 (m, 5H, H_pip_ + -CH(CH_3_)_2_), 3.35–3.08 (m, 6H, -CH_2_N_pip_ + H_pip_), 1.11 (d, *J* = 6.4 Hz, 6H, -CH(CH_3_)_2_); ^13^C-NMR (100.53 MHz, DMSO-*d*_6_) δ (ppm): 165.19, 162.62, 151.82, 139.23, 131.60, 123.57, 121.62, 120.84, 118.29, 114.44, 111.96, 71.11, 67.91, 66.12, 65.74, 63.27, 58.27, 55.40, 52.63, 51.16, 46.82, 46.67, 22.01; HR-MS: C_26_H_37_N_2_O_6_ [M − H]^−^ calculated 473.2646 *m*/*z*, found 473.2640 *m*/*z*.

*1-{2-Hydroxy-3-[(4-isopropoxyethoxybenzoyl)oxy]propyl}-4-(4-methoxyphenyl)piperazinediium dichloride* (**8c**). Yield: 34%; *R*_f_: 0.81 (acetone/toluene 3:1); *R*_f_ (rev.): 0.23 (0.1 M HCl/acetone 3:2); m.p.: 193–195 °C; HPLC pur. 95.60 (254 nm); IR (ATR, cm^−1^): ν(O–H) = 3270, ν(C–H) = 2970, ν(NH^+^) = 2561, ν(C=O) = 1698, ν(arom. C=C) = 1601, ν(C–N) = 1247, ν(C–O–C) = 1179; ^1^H-NMR (399.78 MHz, DMSO-*d*_6_) δ (ppm): 10.74 (bs, 2H, NH^+^_pip_), 7.99 (d, *J* = 8.7 Hz, 2H, H_ArCOO_), 7.07–7.03 (m, 4H, H_ArO_ + H_Ar-pip_), 6.89 (d, *J* = 9.1 Hz, 2H, H_Ar-pip_), 4.50–4.44 (m, 1H, CH_OH_), 4.23–4.21 (m, 2H, COOCH_2_), 4.17–4.14 (m, 2H, ArOCH_2_), 3.76–3.57 (m, 10H, -CH_2_OCH(CH_3_)_2_ + OCH_3_ + H_pip_), 3.45–3.22 (m, 6H, -CH_2_N_pip_ + H_pip_), 1.10 (d, *J* = 5.9 Hz, 6H, -CH(CH_3_)_2_); ^13^C-NMR (100.53 MHz, DMSO-*d_6_*) δ (ppm): 165.24, 162.63, 154.24, 142.82, 131.62, 121.63, 118.41, 114.48, 114.46, 71.13, 67.93, 66.17, 65.76, 63.38, 58.05, 55.28, 52.06, 50.69, 46.96, 46.87, 22.03; HR-MS: C_26_H_37_N_2_O_6_ [M − H]^−^ calculated 473.2646 *m*/*z*, found 473.2641 *m*/*z*.

*1-{2-Hydroxy-3-[(4-propoxyethoxybenzoyl)oxy]propyl}-4-phenylpiperazinediium dichloride* (**9a**). Yield: 32%; *R*_f_: 0.85 (acetone/toluene 3:1); *R*_f_ (rev.): 0.20 (0.1 M HCl/acetone 3:2); m.p.: 197–199 °C; HPLC pur. 96.27% (254 nm); IR (ATR, cm^−1^): ν(O–H) = 3295, ν(C–H) = 2958, ν(NH^+^) = 2351, ν(C=O) = 1702, ν(arom. C=C) = 1601, ν(C–N) = 1250, ν(C–O–C) = 1161; ^1^H-NMR (399.78 MHz, DMSO-*d*_6_) δ (ppm): 10.88 (bs, 2H, NH^+^_pip_), 7.99 (d, *J* = 8.7 Hz, 2H, H_ArCOO_), 7.28–7.24 (m, 2H, H_Ar-pip_), 7.06 (d, *J* = 8.7 Hz, 2H, H_ArO_), 7.03–7.01 (m, 2H, H_Ar-pip_), 6.89–6.85 (m, 1H, H_Ar-pip_), 4.52–4.46 (m, 1H, CH_OH_), 4.23–4.21 (m, 2H, COOCH_2_), 4.19–4.17 (m, 2H, ArOCH_2_), 3.83–3.61 (m, 6H, -CH_2_OPr + H_pip_), 3.44–3.15 (m, 8H, -CH_2_CH_2_CH_3_ + CH_2_N_pip_ + H_pip_), 1.55–1.47 (m, 2H, -CH_2_CH_2_CH_3_), 0.85 (t, *J* = 7.5 Hz, 3H, -CH_2_CH_2_CH_3_); ^13^C-NMR (100.53 MHz, DMSO-*d*_6_) δ (ppm): 165.23, 162.59, 149.42, 131.62, 129.17, 121.66, 120.17, 116.03, 114.44, 72.04, 68.35, 67.58, 66.21, 63.39, 58.16, 52.03, 50.79, 45.38, 45.26, 22.40, 10.50; HR-MS: C_23_H_35_N_2_O_5_ [M − H]^−^ calculated 443.2540 *m*/*z*, found 443.2534 *m*/*z*.

*1-{2-Hydroxy-3-[(4-propoxyethoxybenzoyl)oxy]propyl}-4-(2-methoxyphenyl)piperazinediium dichloride* (**9b**). Yield: 28%; *R*_f_: 0.81 (acetone/toluene 3:1); *R*_f_ (rev.): 0.24 (0.1 M HCl/acetone 3:2); m.p.: 143–146 °C; HPLC pur. 98.29% (254 nm); IR (ATR, cm^−1^): ν(O–H) = 3290, ν(C–H) = 2966, ν(NH^+^) = 2350, ν(C=O) = 1700, ν(arom. C=C) = 1605, ν(C–N) = 1247, ν(C–O–C) = 1170; ^1^H-NMR (399.78 MHz, DMSO-*d*_6_) δ (ppm): 10.70 (bs, 2H, NH^+^_pip_), 7.99 (d, *J* = 8.7 Hz, 2H, H_ArCOO_), 7.07 (d, *J* = 8.7 Hz, 2H, H_ArO_), 7.03–6.89 (m, 4H, H_Ar-pip_), 4.50–4.44 (m, 1H, CH_OH_), 4.23–4.21 (m, 2H, COOCH_2_), 4.20–4.18 (m, 2H, ArOCH_2_-), 3.80 (s, 3H, OCH_3_), 3.73–3.70 (m, 2H, -CH_2_OPr), 3.69–3.46 (m, 4H, H_pip_), 3.41 (t, *J* = 6.6 Hz, 2H, -CH_2_CH_2_CH_3_), 3.32–3.09 (m, 6H, -CH_2_N_pip_ + H_pip_), 1.56–1.47 (m, 2H, -CH_2_CH_2_CH_3_), 0.86 (t, *J* = 7.3 Hz, 3H, -CH_2_CH_2_CH_3_); ^13^C-NMR (100.53 MHz, DMSO-*d*_6_) δ (ppm): 165.19, 162.59, 151.82, 139.21, 131.61, 123.60, 121.65, 120.85, 118.30, 114.43, 111.98, 72.03, 68.34, 67.57, 66.13, 63.29, 58.29, 55.40, 52.61, 51.20, 46.83, 46.69, 22.38, 10.47; HR-MS: C_26_H_37_N_2_O_6_ [M − H]^−^ calculated 473.2646 *m*/*z*, found 473.2642 *m*/*z*.

*1-{2-Hydroxy-3-[(4-propoxyethoxybenzoyl)oxy]propyl}-4-(4-methoxyphenyl)piperazinediium dichloride* (**9c**). Yield: 25%; *R*_f_: 0.81 (acetone/toluene 3:1); *R*_f_ (rev.): 0.22 (0.1 M HCl/acetone 3:2); m.p.: 173–175 °C; HPLC pur. 95.52 (254 nm); IR (ATR, cm^−1^): ν(O–H) = 3279, ν(C–H) = 2934, ν(NH^+^) = 2562, ν(C=O) = 1700, ν(arom. C=C) = 1601, ν(C–N) = 1246, ν(C–O–C) = 1182; ^1^H-NMR (399.78 MHz, DMSO-*d*_6_) δ (ppm): 10.90 (bs, 2H, NH^+^_pip_), 7.99 (d, *J* = 8.7 Hz, 2H, H_ArCOO_), 7.08–7.05 (m, 4H, H_ArO_ + H_Ar-pip_), 6.89 (d, *J* = 9.1 Hz, 2H, H_Ar-pip_), 4.52–4.46 (m, 1H, CH_OH_), 4.23–4.21 (m, 2H, COOCH_2_), 4.19–4.17 (m, 2H, ArOCH_2_), 3.78–3.63 (m, 9H, -CH_2_OPr + OCH_3_ + H_pip_), 3.45–3.22 (m, 8H, -CH_2_CH_2_CH_3_ + -CH_2_N_pip_ + H_pip_), 1.56–1.47 (m, 2H, -CH_2_CH_2_CH_3_), 0.85 (t, *J* = 7.5 Hz, 3H, -CH_2_CH_2_CH_3_); ^13^C-NMR (100.53 MHz, DMSO-*d*_6_) δ (ppm): 165.22, 162.60, 154.49, 142.41, 131.63, 121.65, 118.61, 114.52, 114.44, 72.04, 68.35, 67.57, 66.19, 63.43, 58.05, 55.29, 51.89, 50.65, 47.13, 47.05, 22.40, 10.50; HR-MS: C_26_H_37_N_2_O_6_ [M − H]^−^ calculated 473.2646 *m*/*z*, found 473.2643 *m*/*z*.

### 3.3. Determination of Physicochemical Parameters

The log *k_w_*^7.4^ values of synthetized compounds were measured by a Dionex Ultimate 3000 (Thermo Fisher Scientific) HPLC system controlled through the Chromeleon^®^ Chromatography Data System (version 7.2). The separation was performed on ZORBAX Extend-C_18_ (3.5 µm, 3 × 150 mm) column (Agilent Technologies). Mobile phase consists of 0.02 M 3-morphol, that can be used with addition of 0.15% of *n*-decylamine (pH = 7.4; constituent A) and methanol to which 0.25% of *n*-octanol was added (constituent B). The total flow rate was 0.4 mL/min, the injection volume was 1 μL, and the column temperature was maintained at 25 °C. The detection wavelength of 254 nm was chosen. One milligram of the sample was dissolved in 1 mL of methanol and diluted to the concentration 0.1 mg/mL with 50% methanol. Retention factors of the compound were measured under isocratic conditions in the range 20:80–50:50 (A:B; *v*/*v*), 5 values for each factor in duplicate. Linear regression analysis was performed and log $k_{w}^{7.4}$ values were calculated according to Equation (1).

(1) $$\log k=-S\phi +\log k_{w}$$

where log *k* represents a logarithm of individual isocratic factor, φ is the organic phase concentration, and *S* is a constant derived by linear regression analysis. The retention factor corresponding to the neutral form (log *k_w_*) was estimated from the apparent log $k_{w}^{7.4}$ by using Equation (2).

(2) $$\log k_{w}=\log k_{w}^{app}+\log(1+10^{pKa-pH})$$

The CE experiments were carried out with an Agilent 3D CE (Agilent Technologies) capillary electrophoresis system equipped with an autosampler, automatic injector, photodiode array detector, and an air cooling unit for the capillary. The instrument control and analysis were performed with 3D-CE ChemStation software (Agilent Technologies). Uncoated fused-silica capillary (Agilent Technologies) of 50 µm internal diameter, total length of 33.0 cm and effective length (to the detector) of 24.5 cm was used. The capillary cassette temperature was maintained at 25 °C with air cooling. Three milligrams of the sample were dissolved in 1 mL of 50% methanol and 10 times diluted with appropriate background electrolyte before analysis. Samples were injected hydrodynamically at 40 mbar pressure for 4.0 s. Running voltage was 15 kV of positive polarity. UV detection was performed at 254 nm. As a marker of electroosmotic flow, mesityl oxide was used. The effective mobilities were recorded for BGE pH values in the range of 3.9 to 9.2 (ionic strength equal to 10 mM), 4 runs at 10 different buffer pH values. The effective mobilities (μ*_eff_*) were calculated according to Equation (3).

(3) $$\mu_{eff}=\frac{L_{tot}L_{eff}}{U}(\frac{1}{t_{mig}}-\frac{1}{t_{EOF}})$$

where *t_mig_* is the migration time of the analyte and *t_EOF_* is the migration time of a neutral marker compound that is of the same velocity as the electroosmotic flow (EOF). *L_eff_* is distance from the injection end of capillary to the detector and *L_tot_* is the total length of the capillary over which the voltage *U* was applied. SigmaPlot for Windows version 11.0 (Systat Software GmbH, Erkrath, Germany) was employed for the nonlinear regression analysis of µ*_eff_*/pH relationship having characteristic sigmoidal shape with inflection point indicating p*K*_a_ value.

The pH measurements were taken with a combination glass electrode (HI 1332B, HANNA Instruments, Woonsocket, RI, USA), using a Thermo Orion 370 PerpHect^®^ pH meter (ThermoFisher Scientific).

## 4. Conclusions

In conclusion, nine new potential dual β- and α_1_-blockers were prepared. In addition, three oxiran intermediates (compounds **4**–**6**) have not yet been described in the literature. In this study, the lipophilicity indexes and the p*K*_a_ values have been experimentally determined and compared with software calculated values. Furthermore, we discussed the ADME properties and potential toxicity of prepared compounds by computing the parameters involved in the Lipinski Rule of Five and the log BB parameter describing the possibility of the compound to penetrate through the BBB.

## Figures and Tables

**Table 1:**
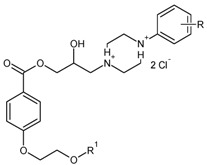
Experimentally determined p*K*_a_ in comparison with calculated values of final compounds.

Comp.	R^1^	R	p*K*_a_^a^ (exp.)	p*K*_a_^b^ (calc.)	p*K*_a_^c^ (calc.)
**7a**	allyl	H	6.47 ± 0.02	6.60	7.70
**7b**	allyl	2-MeO	6.88 ± 0.02	6.70	7.40
**7c**	allyl	4-MeO	6.49 ± 0.02	6.70	7.63
**8a**	isopropyl	H	6.49 ± 0.01	6.60	7.70
**8b**	isopropyl	2-MeO	6.92 ± 0.01	6.70	7.40
**8c**	isopropyl	4-MeO	6.35 ± 0.02	6.70	7.63
**9a**	propyl	H	6.50 ± 0.02	6.60	7.70
**9b**	propyl	2-MeO	6.79 ± 0.02	6.70	7.40
**9c**	propyl	4-MeO	6.39 ± 0.01	6.70	7.63

^a^ Mean and standard deviation of four measurements; ^b^ calculated by ACD Percepta, ver. 2012; ^c^ calculated by MarvinSketch 6.0.6.

**Table 2:**
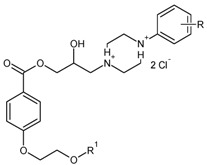
Experimentally determined and calculated values of lipophilicity indexes (log $k_{w}^{7.4}$, log *k_w_*) in comparison with software-calculated values of distribution coefficients (log *D*_7.4_) and lipophilicity (log *P*) of final compounds.

Comp.	R^1^	R	log *k_w_*^7.4^ ^a^	log *D*_7.4_^b^	log *D*_7.4_^c^	log *k_w_*	log *P* ^b^	log *P* ^c^
**7a**	allyl	H	3.97 ± 0.03	3.97	3.15	4.01 ± 0.09	4.03	3.63
**7b**	allyl	2-MeO	3.78 ± 0.02	3.47	3.17	3.89 ± 0.08	3.65	3.47
**7c**	allyl	4-MeO	3.67 ± 0.02	3.40	3.04	3.71 ± 0.08	3.55	3.47
**8a**	isopropyl	H	4.21 ± 0.01	4.03	3.19	4.25 ± 0.05	4.09	3.67
**8b**	isopropyl	2-MeO	4.03 ± 0.02	3.38	3.21	4.15 ± 0.07	3.56	3.51
**8c**	isopropyl	4-MeO	3.90 ± 0.02	3.35	3.08	3.93 ± 0.06	3.50	3.51
**9a**	propyl	H	4.29 ± 0.03	4.01	3.30	4.34 ± 0.10	4.07	3.78
**9b**	propyl	2-MeO	4.11 ± 0.02	3.49	3.32	4.20 ± 0.09	3.67	3.62
**9c**	propyl	4-MeO	3.99 ± 0.02	3.41	3.19	4.03 ± 0.09	3.56	3.62

^a^ Mean and standard deviation of four measurements; ^b^ calculated by ACD Percepta, ver. 2012; ^c^ calculated by MarvinSketch 6.0.6.

**Table 3:** Calculated parameters related to toxicity and absorption, distribution, metabolism, and excretion (ADME).

Comp.	MW ^a^ (<500)	nOHNH (<5)	nON (<10)	nRTB (≤10)	PSA_pH 7.0_^b^ (≤140)	Log BB Class ^c^ (Model 1)	Log BB Class ^c^ (Model 2)
**7a**	440.53	3	7	13	72.67	−0.29	0.32
**7b**	470.56	3	8	14	81.90	−0.61	−0.01
**7c**	470.56	3	8	14	81.90	−0.70	−0.05
**8a**	442.56	3	7	12	72.67	−0.17	0.38
**8b**	472.58	3	8	13	81.90	−0.48	0.06
**8c**	472.58	3	8	13	81.90	−0.59	0.01
**9a**	442.56	3	7	13	72.67	−0.12	0.40
**9b**	472.58	3	8	14	81.90	−0.45	0.07
**9c**	472.58	3	8	14	81.90	−0.54	0.03
**esmolol**	295.37	2	5	10	72.37	−1.37 ^d^	−0.15 ^d^
**flestolol**	327.35	4	7	9	118.26	−3.52 ^d^	−3.17 ^d^
**landiolol**	510.60	3	11	14	132.40	−3.96 ^d^	−3.65 ^d^

^a^ Calculated for free bases by ACD ChemSketch; ^b^ calculated by MarvinSketch; ^c^ calculated from experimental (log *k_w_*) and calculated (PSA) data according to the models generated by Vilar et al. [27]; ^d^ data for calculation were taken from PubChem. MW, molecular weight; nOHNH, number of hydrogen bond donors; nON, number of hydrogen bond acceptors; nRTB, number of rotatable bonds; PSA, polar surface area; log BB, blood–brain barrier penetration.
